# Supplementary material for: Diagnostic testing preferences can help inform future public health response efforts: Global insights from an international survey
Source: PLOS Glob Public Health. 2024 Jul 30;4(7):e0003547. doi: 10.1371/journal.pgph.0003547 (PMC11288416; doi:10.1371/journal.pgph.0003547)
Supplement: S1 Appendix — Table A. Types of tests for the detection of SARS-CoV-21. Table B. Sample types commonly used for the detection of SARS-CoV-2 [supporting images available upon request from the authors]. Table C. Q9—Q11 Keywords and Categories for Analysis. Table D. Geographical data for survey sample. Table E. Number of responses per question. Table F. Most preferred diagnostic testing method in Asia. Table G. Most preferred diagnostic testing method in Europe. Table H. Most preferred diagnostic testing method in Latin America & The Caribbean. Table I. Most preferred diagnostic testing method in North America. Table J. Most preferred diagnostic testing method in Africa. Table K. Most preferred diagnostic testing method in the Middle East. Table L. Most preferred diagnostic testing method in Oceania. Table M. Most preferred diagnostic testing method for children in Asia. Table N. Most preferred diagnostic testing method for children in Europe. Table O. Most preferred diagnostic testing method for children in Latin America & The Caribbean. Table P. Most preferred diagnostic testing method for children in North America. Table Q. Most preferred diagnostic testing method for children in Africa. Table R. Most preferred diagnostic testing method for children in the Middle East. Table S. Most preferred diagnostic testing method for children in Oceania. Table T. Pairwise comparison of preferred sample type for a COVID-19 test by subgroup using Wilcoxon signed-rank test with Bonferroni correction. Table U. Pairwise comparison of preferred overall clinical sample type by subgroup using Wilcoxon signed-rank test with Bonferroni correction. (PDF) [file pgph.0003547.s001.pdf]

S1 APPENDIX
Supplementary Information

Diagnostic testing preferences can help inform future public health response efforts: global insights from an international survey

Leah Salzano[1&], Nithya Narayanan[1&], Emily R. Tobik[1&], Sumaira Akbarzada[1], Yanjun Wu[1], Sarah Megiel[2], Brittany Choate[1,3], Anne L. Wyllie[1*]

[1]Department of Epidemiology of Microbial Diseases, Yale School of Public Health, New Haven, Connecticut, United States of America;

[2]Department of Health Policy and Management, Yale School of Public Health, New Haven, Connecticut, United States of America;

[3]SalivaDirect, Inc., New Haven, Connecticut, United States of America.

[&]These authors contributed equally

*Corresponding Author:

Anne Wyllie

awyllie@gmail.com (ALW)

Table A. Types of tests for the detection of SARS-CoV-2[1]

Type of Test	Description	Role in the Pandemic
Nucleic Acid Amplification Tests (NAATs)	Designed to detect at least one viral RNA gene, used to identify an infection that is current of recent by amplifying the RNA of the virus if any is present in an individual's sample	How diagnostic tests are able to identify individuals infected with SARS-CoV-2<br>Reliable, detect small amounts of SARS-CoV-2, unlikely to give a false positive result<br>High sensitivity and specificity
Antigen	Immunoassays that detect infection of SARS-CoV-2 by detecting the presence of a particular viral antigen	Have enabled at-home testing to become more widespread; rapid test results are beneficial for testing immediately before travel or gathering with others<br>Similar specificity to NAATs, but less sensitive<br>Less expensive than NAATs, may require follow up NAAT to confirm result
Antibody serology testing)	Determines if an individual has detectable antibodies against SARS-CoV-2	A positive test indicates the presence of antibodies to SARS-CoV-2, which means it is possible an individual was recently infected and developed immunity to SARS-CoV-2<br>Help scientists gain an understanding of how the human immune system responds to SARS-CoV-2 and retrospectively assess the attack rate or size of an outbreak

Table B. Sample types commonly used for the detection of SARS-CoV-2[2–4] [supporting images available upon request from the authors]

Sample Type	Collection Method	Pros	Cons	Diagnostic Capability
Anterior-nares (AN) nasal swab	Swab is inserted into a patient's nose and rubbed in a circular motion with moderate pressure against the wall of the nares region.	Can be collected by a healthcare provider or self-collected by the patient.	Lower sensitivity compared to the nasopharyngeal swab.	62.7-90.5% sensitivity[5]
Nasopharyngeal	Swab is inserted into a patient's nostril until it touches the nasopharynx, the upper part of the throat that lies behind the nose.<br>Swab is gently rotated and rubbed against the nasopharynx with the goal of absorbing respiratory secretions and infected cells if they are present.	High sensitivity	Requires trained healthcare professionals in full PPE to collect.<br>Requires special swabs that can be affected by supply chain constraints, making testing more limited and challenging<br>Can induce gagging, coughing, or sneezing, potentially aerosolizing the virus.	74.4-96.5% sensitivity[5]<br>(Reference sample type for US FDA, so trusted source for diagnosing respiratory viruses)
Oral (mouth swab)	Must refrain from food, drink or mouthwash for 20 minutes prior.<br>For 20-30 seconds, swab the inside of each cheek, the upper and lower gums, underneath and top of the tongue, back of the throat, and roof of the mouth.[6]	Can be collected by a healthcare provider or self-collected by the patient		98% sensitivity[7]
Oropharyngeal (throat swab)	Swab is inserted into a patient's mouth and rubbed over both tonsillar pillars and oropharynx.		Requires trained healthcare professional in full PPE to collect.<br>Can induce coughing or gagging.	70.3-94.7% sensitivity[5]
Saliva	Saliva is pooled in the mouth and drooled into a sample tube.	Can be self-collected by the patient.		92-98% sensitivity[8]
Serology test	An antibody or serology test is a blood test that looks for signs of a previous COVID-19 infection. It detects antibodies, which are proteins in the blood that fight off infection.<br>Antibody testing has a lot of promise because it will help us understand the pervasiveness of COVID-19 in our communities.[9]		Must be collected in a doctor's office or clinic, and requires a trained healthcare professional for collection.	Not generally useful for diagnosing COVID-19<br>96% sensitivity based on performance evaluations.[10]

Table C. Q9 - Q11 Keywords and Categories for Analysis

Question	Categories	Keywords
Q9	PCR	‘pcr’
	Lateral Flow Test	'lateral', 'flow', lft'
	Saliva	‘saliva’
	Rapid Antigen Test	'rapid', 'antigen', 'rat'
	Labcorp	'labcorp'
	Multiple	
	Other	
Q10	No preference	'no', 'any'
	Abbott	‘abbott', 'abbot', 'binax'
	Roche	'roche'
	Labcorp	'labcorp'
	Quidel	‘quidel'
	Multiple	
	Other	
Q11	Ease	'ease', 'convenien', 'availabil','comfort', 'easy', 'access'
	Speed	'speed', 'quick', 'fast', ‘time’
	Accuracy	'accur'
	Sensitivity	'sensitivity',
	Reliability	'reliable'
	Multiple	
	Other	

Table D. Geographical data for survey sample

Region	Responses (n)
**Asia**	**166 total**
Bangladesh	3
Bhutan	0
Cambodia	2
China	8
India	35
Indonesia	28
Japan	6
Kazakhstan	3
Malaysia	18

Myanmar	3
Nepal	3
Pakistan	7
Philippines	7
Singapore	6
South Korea	5
Sri Lanka	4
Taiwan	11
Tajikistan	1
Thailand	12
Vietnam	4
**Europe**	**409 total**
Austria	1
Belgium	1
Bosnia & Herzegovina	17
Bulgaria	1
Croatia	1
Czech Republic	2
Denmark	3
Estonia	1
Finland	3
France	2
Georgia	3
Germany	20
Greece	9
Hungary	1
Ireland	8
Italy	21
Latvia	2
Lithuania	1
Luxembourg	0
Netherlands	9
Norway	2
Poland	8
Portugal	3
Romania	3
Russia	14

Serbia	0
Slovakia	1
Slovenia	1
Spain	25
Sweden	3
Switzerland	6
United Kingdom of Great Britain and Northern Ireland	237
**Latin America & The Caribbean**	**186 total**
Argentina	18
Barbados	1
Bolivia	1
Brazil	29
Chile	7
Columbia	12
Costa Rica	1
Ecuador	10
El Salvador	1
French Guiana	1
Guatemala	9
Guyana	2
Honduras	2
Mexico	67
Paraguay	11
Peru	8
Saint Kitts and Nevis	2
Trinidad and Tobago	1
Uruguay	2
Venezuela	1
**North America**	**536 total**
Canada	51
United States	485
**Africa**	**449 total**
Benin	1
Botswana	1
Burkina Faso	1
Cameroon	2
Democratic Republic of the Congo	4

Ethiopia	22
Gabon	1
Ghana	20
Kenya	219
Lesotho	1
Liberia	1
Libya	1
Madagascar	2
Mali	3
Mauritius	1
Morocco	4
Mozambique	2
Nigeria	93
Rwanda	1
Senegal	4
Somalia	1
South Africa	19
South Sudan	1
Sudan	5
Tanzania	1
The Gambia	1
Tunisia	4
Uganda	24
Zambia	6
Zimbabwe	3
**Middle East**	**162 total**
Armenia	3
Azerbaijan	0
Bahrain	3
Egypt	12
Iran	18
Iraq	4
Israel	4
Jordan	2
Kuwait	1
Lebanon (Beirut)	1
Oman	3

Palestine	58
Qatar	3
Saudi Arabia	18
Syria	2
Turkey	20
United Arab Emirates	7
Yemen	3
**Oceania**	**120 total**
Australia	19
New Zealand	99
Papua New Guinea	2

Table E. Number of responses per question

Question	Number of responses received (n)
Question 1	2094
Question 2	2094
Question 3	2094
Question 4	2094
Question 5	2092
Question 6	19
Question 7	150
Question 8	2094
Question 9	2017
Question 10	1903
Question 11	2032
Question 12	2094
Question 13	1781
Question 14	525
Question 15	2094
Question 16	2094
Question 17	2080
Question 18	2081
Question 19	2094
Question 20	2094
Question 21	2021

Table F. Most preferred diagnostic testing method in Asia

Diagnostic Testing Method	Mean Values Of Preference Rank*
Saliva	2.81
Anterior-nares (AN) nasal swab	3.14
Oropharyngeal	3.57
Oral swab	3.08
Nasopharyngeal	3.79
Deep coughing	4.65
**Top Preference by Country**	
Bangladesh	Nasopharyngeal
Bhutan	N/A
Cambodia	Oral Swab/Saliva
China	Saliva
India	AN swab
Indonesia	Nasopharyngeal
Japan	Saliva
Kazakhstan	Deep coughing
Malaysia	Saliva
Myanmar	Nasopharyngeal
Nepal	AN swab
Pakistan	AN swab
Philippines	Saliva
Singapore	Saliva
South Korea	Saliva
Sri Lanka	Saliva
Taiwan	Saliva
Tajikistan	AN swab
Thailand	Oral swab
Vietnam	Oral swab

*lower mean, higher the preference rank

Table G. Most preferred diagnostic testing method in Europe

Diagnostic Testing Method	Mean Values of Preference Rank*
Oral swab	2.58
Anterior-nares (AN) nasal swab	2.49
Saliva	2.98
Oropharyngeal	3.52

Nasopharyngeal	4.61
Deep coughing	4.75
**Top Preference by Country**	
Austria	Nasopharyngeal
Belgium	Saliva
Bosnia & Herzegovina	AN swab
Bulgaria	Saliva
Croatia	Saliva
Czech Republic	Saliva
Denmark	Oral swab
Estonia	AN swab
Finland	AN swab
France	Oral swab
Georgia	Oral swab
Germany	AN swab
Greece	Oral swab
Hungary	Oropharyngeal
Ireland	Oral swab
Italy	Saliva
Latvia	AN swab
Lithuania	AN swab
Luxembourg	Oral swab
Netherlands	Oral swab
Norway	Saliva
Poland	Oral swab
Portugal	Oral swab
Romania	Saliva
Russia	AN swab
Serbia	Saliva
Slovakia	Saliva
Slovenia	AN swab
Spain	Oral swab
Sweden	Saliva
Switzerland	Saliva
United Kingdom	AN swab

*Lower mean, higher the preference rank

Table H. **Most preferred diagnostic testing method in Latin America & The Caribbean**

Diagnostic Testing Method	Mean Values Of Preference Rank*
Saliva	2.61
Oral swab	2.68
Anterior-nares (AN) nasal swab	3.25
Oropharyngeal	3.61
Nasopharyngeal	3.97
Deep coughing	4.68
**Top Preference by Country**	
Argentina	AN swab
Barbados	Saliva
Bolivia	Saliva
Brazil	Saliva
Chile	Oral swab
Columbia	Oral swab
Costa Rica	Nasopharyngeal
Ecuador	Saliva
El Salvador	AN swab
French Guiana	AN swab
Guatemala	AN swab
Guyana	AN swab
Honduras	Oral swab
Mexico	Saliva
Paraguay	Saliva
Peru	Nasopharyngeal
Saint Kitts and Nevis	Oral swab
Trinidad and Tobago	AN swab
Uruguay	Oral swab
Venezuela	Saliva

*Lower mean, higher the preference rank

Table I. **Most preferred diagnostic testing method in North America**

Diagnostic Testing Method	Mean Values Of Preference Rank*
Oral swab	2.28
Anterior-nares (AN) nasal swab	2.35
Saliva	2.51
Oropharyngeal	3.93
Deep coughing	4.97

Nasopharyngeal	2.35
**Top Preference by Country**	
Canada	Oral swab
United States	Oral swab

*Lower mean, higher the preference rank

Table J. **Most preferred diagnostic testing method in Africa**

Diagnostic Testing Method	Mean Values Of Preference Rank*
Oral swab	3.10
Saliva	3.33
Anterior-nares (AN) nasal swab	3.24
Oropharyngeal	3.37
Nasopharyngeal	3.73
Deep coughing	4.01
**Top Preference by Country**	
Benin	Saliva
Botswana	Saliva
Burkina Faso	Nasopharyngeal
Cameroon	Saliva
Democratic Republic of the Congo	Oral swab
Ethiopia	AN swab
Gabon	Oral swab
Ghana	Oral swab
Kenya	AN swab
Lesotho	Saliva
Liberia	Saliva
Libya	AN swab
Madagascar	Oropharyngeal
Mali	Oropharyngeal
Mauritius	Nasopharyngeal
Morocco	AN swab
Mozambique	Saliva
Nigeria	Oral swab
Rwanda	Oral swab
Senegal	Saliva
Somalia	Saliva
South Africa	Oral swab

South Sudan	Nasopharyngeal
Sudan	Oral swab
Tanzania	Saliva
The Gambia	Oral swab
Tunisia	Nasopharyngeal
Uganda	Oral swab
Zambia	Saliva
Zimbabwe	Oral swab

*Lower mean, higher the preference rank

Table K. Most preferred diagnostic testing method in the Middle East

Diagnostic Testing Method	Mean Values Of Preference Rank*
Oral swab	3.15
Saliva	3.36
Anterior-nares (AN) nasal swab	3.09
Oropharyngeal	3.38
Nasopharyngeal	3.58
Deep coughing	4.38
**Top Preference by Country**	
Armenia	AN swab
Azerbaijan	N/A
Bahrain	AN swab
Egypt	Nasopharyngeal
Iran	Oral swab
Iraq	AN swab
Israel	Oral swab
Jordan	Oropharyngeal
Kuwait	Oropharyngeal
Lebanon (Beirut)	Saliva
Oman	Saliva
Palestine	AN swab
Qatar	Oral swab
Saudi Arabia	Oral swab
Syria	AN swab
Turkey	Saliva
United Arab Emirates (UAE)	AN swab
Yemen	Nasopharyngeal

*Lower mean, higher the preference rank

Table L. **Most preferred diagnostic testing method in Oceania**

Diagnostic Testing Method	Mean Values Of Preference Rank*
Saliva	2.19
Oral swab	2.13
Anterior-nares (AN) nasal swab	3.06
Oropharyngeal	3.78
Deep coughing	5.01
Nasopharyngeal	4.92
**Top Preference by Country**	
Australia	Oral swab
New Zealand	Saliva
Papua New Guinea	Nasopharyngeal

*Lower mean, higher the preference rank

Table M. **Most preferred diagnostic testing method for children in Asia**

Diagnostic Testing Method	Responses (%)
Drooling saliva into a small plastic tube (saliva)	30.2%
I do not have children	23.3%
Using a swab to collect a sample from about halfway up your nose (anterior-nares (AN) nasal swab)	12.8%
Using a swab to collect a sample from going all the way through your nose to the back of your throat (nasopharyngeal)	12.8%
Using a swab to collect a sample from just inside your mouth (oral swab)	10.5%
Using a swab to collect a sample from the back of your mouth (oropharyngeal)	8.1%
Deep coughing to collect fluid from in the lungs	2.3%
**Top Preference by Country**	
Bangladesh	Nasopharyngeal
Bhutan	N/A
Cambodia	Deep Coughing
China	Saliva
India	Saliva
Indonesia	Saliva
Japan	Nasopharyngeal
Kazakhstan	Saliva
Malaysia	Saliva
Myanmar	Saliva
Nepal	Saliva
Pakistan	Nasopharyngeal

Philippines	Saliva
Singapore	Saliva
South Korea	Saliva
Sri Lanka	Saliva
Taiwan	Saliva
Tajikistan	Deep Coughing
Thailand	Saliva
Vietnam	Oral Swab

Table N. **Most preferred diagnostic testing method for children in Europe**

Diagnostic Testing Method	Responses (%)
I do not have children	40.7%
Using a swab to collect a sample from about halfway up your nose (anterior-nares (AN) nasal swab)	20.4%
Drooling saliva into a small plastic tube (saliva)	16.7%
Using a swab to collect a sample from just inside your mouth (oral swab)	16.7%
Using a swab to collect a sample from going all the way through your nose to the back of your throat (nasopharyngeal)	3.7%
Using a swab to collect a sample from the back of your mouth (oropharyngeal)	0%
Deep coughing to collect fluid from in the lungs	0%
**Top Preference by Country**	
Austria	Saliva
Belgium	Oral swab
Bosnia & Herzegovina	Nasopharyngeal
Bulgaria	Deep Coughing
Croatia	Saliva
Czech Republic	Saliva
Denmark	Saliva
Estonia	Deep Coughing
Finland	AN swab
France	Oral Swab
Georgia	Oral Swab
Germany	AN swab
Greece	AN swab
Hungary	Deep Coughing
Ireland	Saliva
Italy	Saliva
Latvia	AN swab

Lithuania	AN swab
Luxembourg	N/A
Netherlands	AN swab
Norway	Deep Coughing
Poland	Deep Coughing
Portugal	Oral Swab
Romania	Saliva
Russia	Oral Swab
Serbia	N/A
Slovakia	Saliva
Slovenia	AN swab
Spain	Saliva
Sweden	Nasopharyngeal
Switzerland	Saliva
United Kingdom	AN swab

Table O. Most preferred diagnostic testing method for children in Latin America & The Caribbean

Diagnostic Testing Method	Responses (%)
I do not have children	37.1%
Drooling saliva into a small plastic tube (saliva)	21.3%
Using a swab to collect a sample from just inside your mouth (oral swab)	20.2%
Using a swab to collect a sample from going all the way through your nose to the back of your throat (nasopharyngeal)	13.5%
Using a swab to collect a sample from about halfway up your nose (anterior-nares (AN) nasal swab)	6.7%
Using a swab to collect a sample from the back of your mouth (oropharyngeal)	1.1%
Deep coughing to collect fluid from in the lungs	0%
**Top Preference by Country**	
Argentina	I do not have children
Barbados	AN swab
Bolivia	AN swab
Brazil	Saliva
Chile	Saliva
Columbia	Saliva
Costa Rica	Nasopharyngeal
Ecuador	Nasopharyngeal
El Salvador	Oropharyngeal
French Guiana	Oral Swab

Guatemala	AN swab
Guyana	Oral Swab
Honduras	Saliva
Mexico	Oral Swab
Paraguay	Saliva
Peru	Saliva
Saint Kitts and Nevis	Oral Swab
Trinidad and Tobago	Nasopharyngeal
Uruguay	Saliva
Venezuela	Nasopharyngeal

Table P. **Most preferred diagnostic testing method for children in North America**

Diagnostic Testing Method	Responses (%)
I do not have children	50.5%
Drooling saliva into a small plastic tube (saliva)	19.3%
Using a swab to collect a sample from just inside your mouth (oral swab)	15.8%
Using a swab to collect a sample from about halfway up your nose (anterior-nares (AN) nasal swab)	12.1%
Using a swab to collect a sample from going all the way through your nose to the back of your throat (nasopharyngeal)	1.0%
Using a swab to collect a sample from the back of your mouth (oropharyngeal)	1.0%
Deep coughing to collect fluid from in the lungs	0.21%
**Top Preference by Country**	
Canada	Oral Swab
United States	Saliva

Table Q. **Most preferred diagnostic testing method for children in Africa**

Diagnostic Testing Method	Responses (%)
Drooling saliva into a small plastic tube (saliva)	26.7%
Using a swab to collect a sample from just inside your mouth (oral swab)	21.7%
I do not have children	20.8%
Using a swab to collect a sample from the back of your mouth (oropharyngeal)	13.3%
Using a swab to collect a sample from about halfway up your nose (anterior-nares (AN) nasal swab)	10.8%
Using a swab to collect a sample from going all the way through your nose to the back of your throat (nasopharyngeal)	3.3%
Deep coughing to collect fluid from in the lungs	3.3%
**Top Preference by Country**	
Benin	Saliva

Botswana	Saliva
Burkina Faso	Oropharyngeal
Cameroon	AN swab
Democratic Republic of the Congo	AN swab
Ethiopia	Saliva
Gabon	Oral swab
Ghana	Oral swab
Kenya	Saliva
Lesotho	Saliva
Liberia	Saliva
Libya	AN swab
Madagascar	Saliva
Mali	Oral swab
Mauritius	Nasopharyngeal
Morocco	Saliva
Mozambique	Saliva
Nigeria	Saliva
Rwanda	Oral swab
Senegal	Saliva
Somalia	AN swab
South Africa	Oral swab
South Sudan	Oral swab
Sudan	Oral swab
Tanzania	Saliva
The Gambia	Oral swab
Tunisia	Oral swab
Uganda	Saliva
Zambia	Saliva
Zimbabwe	Deep coughing

Table R. Most preferred diagnostic testing method for children in the Middle East

Diagnostic Testing Method	Responses (%)
Drooling saliva into a small plastic tube (saliva)	19.3%
Using a swab to collect a sample from just inside your mouth (oral swab)	19.3%
I do not have children	30.7%
Using a swab to collect a sample from the back of your mouth (oropharyngeal)	7.3%
Using a swab to collect a sample from about halfway up your nose (anterior-nares (AN) nasal swab)	10.0%

Using a swab to collect a sample from going all the way through your nose to the back of your throat (nasopharyngeal)	13.3%
Deep coughing to collect fluid from in the lungs	0%
**Top Preference by Country**	
Armenia	AN swab
Azerbaijan	N/A
Bahrain	AN swab
Egypt	Oropharyngeal
Iran	Saliva
Iraq	AN swab
Israel	Oral swab
Jordan	Saliva
Kuwait	Oral swab
Lebanon (Beirut)	Oral swab
Oman	Saliva
Palestine	Oral swab
Qatar	Saliva
Saudi Arabia	Saliva
Syria	Deep coughing
Turkey	Saliva
United Arab Emirates (UAE)	Saliva
Yemen	Nasopharyngeal

Table S. **Most preferred diagnostic testing method for children in Oceania**

Diagnostic Testing Method	Responses (%)
Drooling saliva into a small plastic tube (saliva)	21.0%
Using a swab to collect a sample from just inside your mouth (oral swab)	15.2%
Using a swab to collect a sample from about halfway up your nose (anterior-nares (AN) nasal swab)	7.6%
Using a swab to collect a sample from going all the way through your nose to the back of your throat (nasopharyngeal)	2.0%
Using a swab to collect a sample from the back of your mouth (oropharyngeal)	0.9%
Deep coughing to collect fluid from in the lungs	0%
I do not have children	53.3%
**Top Preference by Country**	
Australia	Saliva
New Zealand	Saliva
Papua New Guinea	Nasopharyngeal

Table T. Pairwise comparison of preferred sample type for a COVID-19 test by subgroup using Wilcoxon signed-rank test with Bonferroni correction

**Overall**	Group 1	Group 2	P-value
	Coughing	Nasal	8.91E-153
	Coughing	NP	0.000363
	Coughing	OP	2.58E-82
	Coughing	Oral	1.28E-203
	Coughing	Saliva	2.92E-190
	Nasal	NP	1.47E-143
	Nasal	OP	4.12E-65
	Nasal	Oral	0.027
	Nasal	Saliva	1
	NP	OP	2.34E-60
	NP	Oral	1.78E-120
	NP	Saliva	9.42E-77
	OP	Oral	5.25E-86
	OP	Saliva	4.23E-38
	Oral	Saliva	0.0000474
**Gender**			
Female	Coughing	Nasal	1.26E-107
	Coughing	NP	0.016
	Coughing	OP	1.11E-57
	Coughing	Oral	1.41E-134
	Coughing	Saliva	1.26E-122
	Nasal	NP	5.24E-102
	Nasal	OP	1.46E-52
	Nasal	Oral	0.194
	Nasal	Saliva	0.158
	NP	OP	6.99E-44
	NP	Oral	4.46E-85
	NP	Saliva	2.41E-53
	OP	Oral	1.78E-65
	OP	Saliva	2.31E-24
	Oral	Saliva	6.69E-08
Male	Coughing	Nasal	6.99E-44
	Coughing	NP	0.07
	Coughing	OP	2.13E-25
	Coughing	Oral	1.66E-64
	Coughing	Saliva	3.86E-65
	Nasal	NP	2.04E-39
	Nasal	OP	1.95E-14
	Nasal	Oral	0.966
	Nasal	Saliva	1

	NP	OP	1.36E-16
	NP	Oral	1.84E-33
	NP	Saliva	5.26E-24
	OP	Oral	2.9E-21
	OP	Saliva	6.68E-14
	Oral	Saliva	1
Non-binary	Coughing	Nasal	0.008
	Coughing	NP	1
	Coughing	OP	0.588
	Coughing	Oral	0.000639
	Coughing	Saliva	0.003
	Nasal	NP	0.013
	Nasal	OP	0.024
	Nasal	Oral	1
	Nasal	Saliva	1
	NP	OP	0.447
	NP	Oral	0.003
	NP	Saliva	0.101
	OP	Oral	0.015
	OP	Saliva	1
	Oral	Saliva	1
Prefer not to respond	Coughing	Nasal	0.198
	Coughing	NP	1
	Coughing	OP	0.199
	Coughing	Oral	0.198
	Coughing	Saliva	0.204
	Nasal	NP	0.204
	Nasal	OP	0.605
	Nasal	Oral	1
	Nasal	Saliva	1
	NP	OP	0.206
	NP	Oral	0.179
	NP	Saliva	0.189
	OP	Oral	1
	OP	Saliva	1
	Oral	Saliva	1
**Age**			
18 - 24	Coughing	Nasal	1.23E-15
	Coughing	NP	0.004
	Coughing	OP	1.23E-07
	Coughing	Oral	3.78E-13
	Coughing	Saliva	2.34E-09
	Nasal	NP	5.64E-10
	Nasal	OP	0.00000562
	Nasal	Oral	0.675
	Nasal	Saliva	0.012

	NP	OP	0.676
	NP	Oral	0.005
	NP	Saliva	1
	OP	Oral	0.023
	OP	Saliva	1
	Oral	Saliva	0.601
25 - 34	Coughing	Nasal	2.26E-30
	Coughing	NP	0.212
	Coughing	OP	1.07E-17
	Coughing	Oral	5.52E-45
	Coughing	Saliva	1.01E-40
	Nasal	NP	1.9E-27
	Nasal	OP	1.03E-10
	Nasal	Oral	1
	Nasal	Saliva	1
	NP	OP	7.9E-11
	NP	Oral	3.49E-22
	NP	Saliva	1.2E-11
	OP	Oral	8.78E-15
	OP	Saliva	0.000104
	Oral	Saliva	0.061
35 - 44	Coughing	Nasal	4.8E-33
	Coughing	NP	1
	Coughing	OP	6.72E-15
	Coughing	Oral	7.52E-48
	Coughing	Saliva	7.08E-49
	Nasal	NP	5.2E-32
	Nasal	OP	3.98E-15
	Nasal	Oral	0.018
	Nasal	Saliva	0.819
	NP	OP	5.3E-12
	NP	Oral	1.81E-29
	NP	Saliva	3.27E-21
	OP	Oral	2.49E-25
	OP	Saliva	5.41E-16
	Oral	Saliva	1
45 - 54	Coughing	Nasal	3.87E-30
	Coughing	NP	1
	Coughing	OP	8.42E-15
	Coughing	Oral	1.59E-41
	Coughing	Saliva	4.36E-38
	Nasal	NP	2.11E-33
	Nasal	OP	6.21E-16
	Nasal	Oral	0.058
	Nasal	Saliva	1
	NP	OP	1.34E-19
	NP	Oral	5.8E-32

	NP	Saliva	2.15E-23
	OP	Oral	4.65E-24
	OP	Saliva	2.14E-12
	Oral	Saliva	1
55 - 64	Coughing	Nasal	4.04E-28
	Coughing	NP	0.459
	Coughing	OP	7.34E-20
	Coughing	Oral	1.17E-37
	Coughing	Saliva	1.1E-37
	Nasal	NP	1.07E-25
	Nasal	OP	8.71E-11
	Nasal	Oral	0.13
	Nasal	Saliva	1
	NP	OP	3.72E-15
	NP	Oral	1.48E-25
	NP	Saliva	1.78E-19
	OP	Oral	3.68E-17
	OP	Saliva	1.29E-09
	Oral	Saliva	1
65+	Coughing	Nasal	5.46E-19
	Coughing	NP	1
	Coughing	OP	4.02E-12
	Coughing	Oral	2.85E-22
	Coughing	Saliva	1.26E-20
	Nasal	NP	5.8E-18
	Nasal	OP	1.56E-11
	Nasal	Oral	1
	Nasal	Saliva	0.055
	NP	OP	6.39E-11
	NP	Oral	1.31E-15
	NP	Saliva	4.9E-11
	OP	Oral	5.66E-10
	OP	Saliva	0.009
	Oral	Saliva	0.002
**Region**			
Africa	Coughing	Nasal	3.78E-07
	Coughing	NP	0.551
	Coughing	OP	4.48E-07
	Coughing	Oral	3.72E-15
	Coughing	Saliva	8.76E-11
	Nasal	NP	0.000034
	Nasal	OP	1
	Nasal	Oral	1
	Nasal	Saliva	1
	NP	OP	0.003
	NP	Oral	0.0000471

	NP	Saliva	0.146
	OP	Oral	0.037
	OP	Saliva	1
	Oral	Saliva	0.166
Asia	Coughing	Nasal	8.56E-09
	Coughing	NP	0.003
	Coughing	OP	0.00000156
	Coughing	Oral	4.94E-12
	Coughing	Saliva	3.75E-14
	Nasal	NP	0.001
	Nasal	OP	0.129
	Nasal	Oral	1
	Nasal	Saliva	1
	NP	OP	1
	NP	Oral	0.01
	NP	Saliva	0.013
	OP	Oral	0.03
	OP	Saliva	0.011
	Oral	Saliva	1
Europe	Coughing	Nasal	3.15E-48
	Coughing	NP	1
	Coughing	OP	1.82E-24
	Coughing	Oral	6.4E-52
	Coughing	Saliva	4.94E-46
	Nasal	NP	2.92E-47
	Nasal	OP	5.7E-23
	Nasal	Oral	1
	Nasal	Saliva	0.000379
	NP	OP	3.21E-24
	NP	Oral	2.52E-36
	NP	Saliva	1.78E-21
	OP	Oral	1.15E-18
	OP	Saliva	0.0000748
	Oral	Saliva	0.0000108
Latin America and the Caribbean	Coughing	Nasal	5.43E-10
	Coughing	NP	0.023
	Coughing	OP	0.00000024
	Coughing	Oral	2.56E-18
	Coughing	Saliva	3E-20
	Nasal	NP	0.000284
	Nasal	OP	0.135
	Nasal	Oral	0.002
	Nasal	Saliva	0.05
	NP	OP	0.094
	NP	Oral	0.00000105
	NP	Saliva	0.0000234
	OP	Oral	1.64E-08

	OP	Saliva	0.00000248
	Oral	Saliva	1
Middle East	Coughing	Nasal	0.00000582
	Coughing	NP	0.012
	Coughing	OP	0.000042
	Coughing	Oral	7.84E-08
	Coughing	Saliva	3.56E-07
	Nasal	NP	0.13
	Nasal	OP	1
	Nasal	Oral	1
	Nasal	Saliva	1
	NP	OP	1
	NP	Oral	0.891
	NP	Saliva	1
	OP	Oral	1
	OP	Saliva	1
	Oral	Saliva	1
North America	Coughing	Nasal	1.62E-73
	Coughing	NP	1
	Coughing	OP	8.34E-32
	Coughing	Oral	1.74E-83
	Coughing	Saliva	1.51E-77
	Nasal	NP	8.73E-79
	Nasal	OP	1.35E-56
	Nasal	Oral	1
	Nasal	Saliva	1
	NP	OP	1.2E-37
	NP	Oral	5.24E-70
	NP	Saliva	3.62E-58
	OP	Oral	1.02E-60
	OP	Saliva	1.7E-37
	Oral	Saliva	0.057
Oceania	Coughing	Nasal	3.81E-13
	Coughing	NP	1
	Coughing	OP	8.2E-09
	Coughing	Oral	6.82E-19
	Coughing	Saliva	1.14E-18
	Nasal	NP	5.79E-13
	Nasal	OP	0.000752
	Nasal	Oral	0.00000566
	Nasal	Saliva	0.004
	NP	OP	7.86E-09
	NP	Oral	8.54E-16
	NP	Saliva	1.98E-14
	OP	Oral	5.07E-15
	OP	Saliva	1.89E-10
	Oral	Saliva	1

**Education**			
Bachelor's degree	Coughing	Nasal	1.38E-36
	Coughing	NP	1
	Coughing	OP	2.64E-20
	Coughing	Oral	9.54E-49
	Coughing	Saliva	1.87E-46
	Nasal	NP	1.1E-36
	Nasal	OP	4.98E-15
	Nasal	Oral	1
	Nasal	Saliva	1
	NP	OP	7.32E-19
	NP	Oral	1.08E-30
	NP	Saliva	1.76E-21
	OP	Oral	1.74E-21
	OP	Saliva	9.68E-09
	Oral	Saliva	0.059
Graduate degree	Coughing	Nasal	5.69E-82
	Coughing	NP	0.053
	Coughing	OP	1.72E-42
	Coughing	Oral	1.2E-120
	Coughing	Saliva	8.26E-117
	Nasal	NP	3.73E-81
	Nasal	OP	2.42E-37
	Nasal	Oral	0.0000097
	Nasal	Saliva	0.214
	NP	OP	2.43E-35
	NP	Oral	7.65E-78
	NP	Saliva	1.32E-53
	OP	Oral	3.99E-64
	OP	Saliva	5.43E-37
	Oral	Saliva	1
Some college, no degree	Coughing	Nasal	1.47E-16
	Coughing	NP	0.094
	Coughing	OP	3.68E-09
	Coughing	Oral	6.88E-16
	Coughing	Saliva	2.19E-12
	Nasal	NP	5.97E-12
	Nasal	OP	0.00000234
	Nasal	Oral	1
	Nasal	Saliva	0.016
	NP	OP	0.004
	NP	Oral	0.00000333
	NP	Saliva	0.032
	OP	Oral	0.005
	OP	Saliva	1
	Oral	Saliva	0.214

**Race**			
African	Coughing	Nasal	1.52E-11
	Coughing	NP	1
	Coughing	OP	1.72E-08
	Coughing	Oral	2.53E-21
	Coughing	Saliva	8.7E-17
	Nasal	NP	2.45E-13
	Nasal	OP	0.019
	Nasal	Oral	1
	Nasal	Saliva	1
	NP	OP	0.00000166
	NP	Oral	2.19E-10
	NP	Saliva	0.000107
	OP	Oral	0.000057
	OP	Saliva	1
	Oral	Saliva	0.334
South Asian	Coughing	Nasal	6.16E-13
	Coughing	NP	0.00018
	Coughing	OP	3.26E-09
	Coughing	Oral	5.67E-14
	Coughing	Saliva	2.42E-11
	Nasal	NP	0.0004
	Nasal	OP	0.003
	Nasal	Oral	1
	Nasal	Saliva	1
	NP	OP	1
	NP	Oral	0.058
	NP	Saliva	0.984
	OP	Oral	0.123
	OP	Saliva	1
	Oral	Saliva	1
East Asian	Coughing	Nasal	5.07E-09
	Coughing	NP	1
	Coughing	OP	0.007
	Coughing	Oral	3.9E-10
	Coughing	Saliva	3.92E-12
	Nasal	NP	4.52E-09
	Nasal	OP	0.00000674
	Nasal	Oral	1
	Nasal	Saliva	1
	NP	OP	0.014
	NP	Oral	8.16E-07
	NP	Saliva	7.68E-07
	OP	Oral	0.0000148
	OP	Saliva	0.0000574
	Oral	Saliva	1
Latino/Hispanic	Coughing	Nasal	2.66E-08

	Coughing	NP	0.258
	Coughing	OP	0.0000069
	Coughing	Oral	6.72E-19
	Coughing	Saliva	3.54E-19
	Nasal	NP	0.00048
	Nasal	OP	0.225
	Nasal	Oral	0.000021
	Nasal	Saliva	0.008
	NP	OP	0.044
	NP	Oral	1.22E-08
	NP	Saliva	0.0000101
	OP	Oral	1.02E-09
	OP	Saliva	8.49E-07
	Oral	Saliva	1
Middle Eastern	Coughing	Nasal	0.021
	Coughing	NP	1
	Coughing	OP	0.011
	Coughing	Oral	0.0000177
	Coughing	Saliva	0.000354
	Nasal	NP	0.481
	Nasal	OP	1
	Nasal	Oral	1
	Nasal	Saliva	1
	NP	OP	1
	NP	Oral	0.144
	NP	Saliva	1
	OP	Oral	0.544
	OP	Saliva	1
	Oral	Saliva	0.654
Other	Coughing	Nasal	0.0000138
	Coughing	NP	0.861
	Coughing	OP	0.002
	Coughing	Oral	3.81E-09
	Coughing	Saliva	3.1E-10
	Nasal	NP	0.000332
	Nasal	OP	0.113
	Nasal	Oral	0.279
	Nasal	Saliva	0.334
	NP	OP	0.049
	NP	Oral	0.0000357
	NP	Saliva	0.000252
	OP	Oral	0.0000393
	OP	Saliva	0.002
	Oral	Saliva	1
Prefer not to say	Coughing	Nasal	0.0000752
	Coughing	NP	0.012
	Coughing	OP	0.001

	Coughing	Oral	0.002
	Coughing	Saliva	0.004
	Nasal	NP	0.001
	Nasal	OP	0.000262
	Nasal	Oral	0.107
	Nasal	Saliva	0.082
	NP	OP	1
	NP	Oral	1
	NP	Saliva	1
	OP	Oral	1
	OP	Saliva	1
	Oral	Saliva	1

Table U. Pairwise comparison of preferred overall clinical sample type by subgroup using Wilcoxon signed-rank test with Bonferroni correction

	Group1	Group2	P-value
**Overall**	Blood	Saliva	1.2E-165
	Blood	Urine	5.25E-09
	Saliva	Urine	1.2E-184
**Gender**			
Female	Blood	Saliva	2.11E-90
	Blood	Urine	0.000636
	Saliva	Urine	1.6E-103
Male	Blood	Saliva	9.06E-74
	Blood	Urine	4.2E-06
	Saliva	Urine	2.51E-79
Non-binary	Blood	Saliva	0.009
	Blood	Urine	1
	Saliva	Urine	0.005
Prefer not to respond	Blood	Saliva	0.057
	Blood	Urine	1
	Saliva	Urine	0.025
**Age**			
18 - 24	Blood	Saliva	5.91E-05
	Blood	Urine	2.48E-05
	Saliva	Urine	6.75E-17
25 - 34	Blood	Saliva	4.02E-36
	Blood	Urine	0.513
	Saliva	Urine	6.09E-44
35 - 44	Blood	Saliva	2.6E-48
	Blood	Urine	0.000612
	Saliva	Urine	2.54E-56
45 - 54	Blood	Saliva	1.24E-39
	Blood	Urine	1.83E-07
	Saliva	Urine	2.8E-40
55 - 64	Blood	Saliva	4.29E-35
	Blood	Urine	1.88E-08
	Saliva	Urine	1.4E-25
65+	Blood	Saliva	1.46E-12
	Blood	Urine	0.004
	Saliva	Urine	6.66E-12
**Region**			
Africa	Blood	Saliva	1.59E-11
	Blood	Urine	1
	Saliva	Urine	6.06E-18
Asia	Blood	Saliva	2.67E-12

	Blood	Urine	1
	Saliva	Urine	8.88E-15
Europe	Blood	Saliva	4.65E-39
	Blood	Urine	1.67E-05
	Saliva	Urine	1.39E-39
Latin America and the Caribbean	Blood	Saliva	7.89E-18
	Blood	Urine	1
	Saliva	Urine	2.55E-24
Middle East	Blood	Saliva	8.07E-06
	Blood	Urine	0.000303
	Saliva	Urine	1.54E-15
North America	Blood	Saliva	9.72E-78
	Blood	Urine	2.02E-19
	Saliva	Urine	1.28E-70
Oceania	Blood	Saliva	3.42E-16
	Blood	Urine	0.019
	Saliva	Urine	1.43E-16
**Education**			
Bachelor's degree	Blood	Saliva	2.19E-37
	Blood	Urine	1
	Saliva	Urine	8.73E-50
Graduate degree	Blood	Saliva	4.2E-117
	Blood	Urine	4.86E-16
	Saliva	Urine	1.9E-118
Some college, no degree	Blood	Saliva	6.21E-05
	Blood	Urine	0.549
	Saliva	Urine	1.95E-09
**Race**			
African	Blood	Saliva	6.93E-19
	Blood	Urine	0.831
	Saliva	Urine	5.82E-23
South Asian	Blood	Saliva	1.49E-06
	Blood	Urine	1
	Saliva	Urine	6.21E-12
East Asian	Blood	Saliva	6.24E-10
	Blood	Urine	1
	Saliva	Urine	6.42E-13
Latino/Hispanic	Blood	Saliva	8.7E-20
	Blood	Urine	0.465
	Saliva	Urine	5.28E-25
Middle Eastern	Blood	Saliva	3.69E-07
	Blood	Urine	0.1
	Saliva	Urine	4.74E-13
White/Caucasian	Blood	Saliva	3.3E-115
	Blood	Urine	4.62E-19

	Saliva	Urine	5.3E-113
Other	Blood	Saliva	3.48E-08
	Blood	Urine	0.513
	Saliva	Urine	1.26E-06
Prefer not to say	Blood	Saliva	0.292
	Blood	Urine	0.279
	Saliva	Urine	0.001

REFERENCES

1. CDC. Guidance for healthcare workers about COVID-19 (SARS-CoV-2) testing. *Centers for Disease Control and Prevention* https://www.cdc.gov/coronavirus/2019-ncov/hcp/testing.html (2023).
2. Marty, F. M., Chen, K. & Verrill, K. A. How to obtain a nasopharyngeal swab specimen. *N. Engl. J. Med.* 382, e76 (2020).
3. How to collect an anterior nasal swab specimen for COVID-19 testing. https://www.cdc.gov/coronavirus/2019-ncov/downloads/testing/QuickieAnteriorSwab.pdf.
4. CDC. Overview of Testing for SARS-CoV-2, the virus that causes COVID-19. *Centers for Disease Control and Prevention* https://www.cdc.gov/coronavirus/2019-ncov/hcp/testing-overview.html (2023).
5. Berenger, B. M., Fonseca, K., Schneider, A. R., Hu, J. & Zelyas, N. Sensitivity of nasopharyngeal, nasal and throat swab for the detection of SARS-CoV-2. *bioRxiv* (2020) doi:10.1101/2020.05.05.20084889.
6. Santos-Longhurst, A. Mouth swab drug test: What to expect. *Healthline* https://www.healthline.com/health/mouth-swab-drug-test (2020).
7. COVID-19 diagnostic genetic test. *Machaon Diagnostics* https://www.machaondiagnostics.com/test/covid-19-diagnostic-genetic-test/ (2023).
8. Gagnon, F. *et al.* Nasopharyngeal swabs vs. saliva sampling for SARS-CoV-2 detection: A cross-sectional survey of acceptability for caregivers and children after experiencing both methods. *PLoS One* 17, e0270929 (2022).
9. COVID-19 antibody testing: What you need to know. https://www.uclahealth.org/news/covid-19-antibody-testing-what-you-need-to-know.
10. CDC. Interim guidelines for COVID-19 antibody testing. *Centers for Disease Control and Prevention* https://www.cdc.gov/coronavirus/2019-ncov/hcp/testing/antibody-tests-guidelines.html (2023).
