# Supplementary material for: Diagnostic testing preferences can help inform future public health response efforts: Global insights from an international survey
Source: PLOS Glob Public Health. 2024 Jul 30;4(7):e0003547. doi: 10.1371/journal.pgph.0003547 (PMC11288416; doi:10.1371/journal.pgph.0003547)
Supplement: Attachment — Submitted filename: 2024_test-pref_reviewers.docx [file pgph.0003547.s003.docx]

**Journal Requirements**

**1. Please review your reference list to ensure that it is complete and correct. If you have cited papers that have been retracted, please include the rationale for doing so in the manuscript text, or remove these references and replace them with relevant current references. Any changes to the reference list should be mentioned in the rebuttal letter that accompanies your revised manuscript. If you need to cite a retracted article, indicate the article’s retracted status in the References list and also include a citation and full reference for the retraction notice.**

*Response:* We have reviewed all of our references to ensure they are still in good standing. There was one publishing correction for which we provide an updated link and we have updated one reference from its preprint to its peer-reviewed citation.

**2. Please send a completed 'Competing Interests' statement, including any COIs declared by your co-authors. If you have no competing interests to declare, please state "The authors have declared that no competing interests exist". Otherwise please declare all competing interests beginning with the statement "I have read the journal's policy and the authors of this manuscript have the following competing interests:"**

*Response:* I have read the journal's policy and the authors of this manuscript have the following competing interests: ALW has received consulting and/or advisory board fees from Pfizer, Merck, Diasorin, PPS Health, Co-Diagnostics, and Global Diagnostic Systems for work unrelated to this project, and is Principal Investigator on research grants from Merck, NIH RADx UP and SalivaDirect, Inc. to Yale University and from NIH RADx, Balvi.io and Shield T3 to SalivaDirect, Inc. In addition, BC is an employee and ALW is a Board Member of the non-profit organization, SalivaDirect, Inc. All other co-authors declare no potential conflict of interest. All other authors have declared that no competing interests exist.

**3. Please amend your detailed Financial Disclosure statement. This is published with the article. It must therefore be completed in full sentences and contain the exact wording you wish to be published.**

A. **State the initials, alongside each funding source, of each author to receive each grant.**
   *Response:* We have updated this.
B. **State what role the funders took in the study. If the funders had no role in your study, please state: “The funders had no role in study design, data collection and analysis, decision to publish, or preparation of the manuscript.”**
   *Response:* We have updated this.
C. **If any authors received a salary from any of your funders, please state which authors and which funders.**

*Response:* All salaries were paid by Yale.

**If you did not receive any funding for this study, please simply state: “The authors received no specific funding for this work.**

*Response:* We have updated the funding statement to report that the grant that supported the study was awarded to ALW. We have included the statement regarding the funders as they had no role in the study. BC is currently an employee of SalivaDirect, Inc. but worked on this study while an employee of Yale University, receiving salary from Yale.

**4. Please provide separate figure files in .tif or .eps format only and remove any figures embedded in your manuscript file. Please also ensure all files are under our size limit of 10MB.**

**For more information about figure files please see our guidelines:**

[**https://journals.plos.org/globalpublichealth/s/figures**](https://journals.plos.org/globalpublichealth/s/figures)
[**https://journals.plos.org/globalpublichealth/s/figures#loc-file-requirement**](https://journals.plos.org/globalpublichealth/s/figures#loc-file-requirement)

*Response:* We have updated this, accordingly.

**5. We notice that your supplementary files are included in the manuscript file. Please remove them and upload them with the file type 'Supporting Information'. Please ensure that each Supporting Information file has a legend listed in the manuscript after the references list.**

*Response:* We apologize for this oversight and have remedied this.

**6. Some material included in your submission may be copyrighted. According to PLOS’s copyright policy, authors who use figures or other material (e.g., graphics, clipart, maps) from another author or copyright holder must demonstrate or obtain permission to publish this material under the Creative Commons Attribution 4.0 International (CC BY 4.0) License used by PLOS journals. Please closely review the details of PLOS’s copyright requirements here: PLOS Licenses and Copyright. If you need to request permissions from a copyright holder, you may use PLOS's Copyright Content Permission form.**

**Please respond directly to this email or email the journal office and provide any known details concerning your material's license terms and permissions required for reuse, even if you have not yet obtained copyright permissions or are unsure of your material's copyright compatibility.**

*Response:* The world map that is presented in our manuscript was generated from an openly available source: Canva Pro. As a Canva Pro graphic, this map is available for use by any subscribing Canva Pro members. We have updated the figure, noting Canva Pro as the source base layer. Additionally, we have uploaded supporting documentation including an invoice showing a current CanvaPro subscription, a screenshot to document the graphic being available in Canva Pro. In addition, a link to Canva's terms of use are available [here](https://www.canva.com/policies/terms-of-use/).

**Potential Copyright Issues:**

**Fig 1: please (a) provide a direct link to the base layer of the map (i.e., the country or region border shape) and ensure this is also included in the figure legend; and (b) provide a link to the terms of use / license information for the base layer image or shapefile. We cannot publish proprietary or copyrighted maps (e.g. Google Maps, Mapquest) and the terms of use for your map base layer must be compatible with our CC-BY 4.0 license.**

***Note: if you created the map in a software program like R or ArcGIS, please locate and indicate the source of the basemap shapefile onto which data has been plotted.***

**If your map was obtained from a copyrighted source please amend the figure so that the base map used is from an openly available source. Alternatively, please provide explicit written permission from the copyright holder granting you the right to publish the material under our CC-BY 4.0 license.**

**Please note that the following CC BY licenses are compatible with PLOS license: CC BY 4.0, CC BY 2.0 and CC BY 3.0, meanwhile such licenses as CC BY-ND 3.0 and others are not compatible due to additional restrictions.**

**If you are unsure whether you can use a map or not, please do reach out and we will be able to help you. The following websites are good examples of where you can source open access or public domain maps:
* U.S. Geological Survey (USGS) - All maps are in the public domain. (**[**http://www.usgs.gov**](http://www.usgs.gov/)**)
* PlaniGlobe - All maps are published under a Creative Commons license so please cite “PlaniGlobe,** [**http://www.planiglobe.com**](http://www.planiglobe.com/)**, CC BY 2.0” in the image credit after the caption. (**[**http://www.planiglobe.com/?lang=enl**](http://www.planiglobe.com/?lang=enl)**)
* Natural Earth - All maps are public domain. (**[**http://www.naturalearthdata.com/about/terms-of-use/**](http://www.naturalearthdata.com/about/terms-of-use/)**)**

*Response:* The world map that is presented in our manuscript was generated from an openly available source: Canva Pro. As a Canva Pro graphic, this map is available for use by any subscribing Canva Pro members. We have updated the figure, noting Canva Pro as the source base layer. Additionally, we have uploaded supporting documentation including an invoice showing a current CanvaPro subscription, a screenshot to document the graphic being available in Canva Pro. In addition, a link to Canva's terms of use are available [here](https://www.canva.com/policies/terms-of-use/).

**Additional Editor Comments (if provided):**
Please ensure the following points raised by reviewers are adequately addressed in the revision/rebuttal:

1. **Clarification and discussion of sampling strategy which may impact the finding of the survey (reviewer 2).**

*Response:* We appreciate these points and have added clarification throughout the paper on the sampling strategy, and provided further detail in the discussion section on the limitations that our sampling method may have posed.

2. **Around the use of the term "saliva", "oral sample" and "oral swab", they do not appear to be properly defined in the survey questions and there are questions from reviewers whether responders understood the implication of these term. Please define them clearly in the manuscript and outline the researchers' attempt to clarify this in the survey. Finally, it is important to discuss the limitations of this accordingly as it is one of the primary purpose of the study.**
   *Response:* We hope our edits in response to the comments from the reviewers have improved the clarity regarding this. We were sure to update the methods section to better reflect the survey itself to aid the reader in understanding the questions that were asked without having to refer to the survey itself. Each of the sample types were depicted and explained in a figure in every survey and then again in the first question under the figure. We have also corrected the “Awareness of sample types” and “Sample type preference” sections of the results where we include and explain these to properly quote the sample type definitions that were provided - we had the sample type outside of the quote of the description, when in the survey it was also part of that description.

**Comments to the Author

1. Does this manuscript meet PLOS Global Public Health’s** [**publication criteria**](https://nam12.safelinks.protection.outlook.com/?url=https%3A%2F%2Fjournals.plos.org%2Fglobalpublichealth%2Fs%2Fcriteria-for-publication&data=05%7C02%7Canne.wyllie%40yale.edu%7Cc1f0618f86cc4cb8b50408dc58dcb506%7Cdd8cbebb21394df8b4114e3e87abeb5c%7C0%7C0%7C638482952158799321%7CUnknown%7CTWFpbGZsb3d8eyJWIjoiMC4wLjAwMDAiLCJQIjoiV2luMzIiLCJBTiI6Ik1haWwiLCJXVCI6Mn0%3D%7C0%7C%7C%7C&sdata=31hLVbtUqkBzUV6MkzXV8TkroUkmGLPyhKb4XMtHHUg%3D&reserved=0)**?** Is the manuscript technically sound, and do the data support the conclusions? The manuscript must describe methodologically and ethically rigorous research with conclusions that are appropriately drawn based on the data presented.

Reviewer #1: Yes

Reviewer #2: Yes

**2. Has the statistical analysis been performed appropriately and rigorously?**

Reviewer #1: No

Reviewer #2: Yes

**3. Have the authors made all data underlying the findings in their manuscript fully available?**
Reviewer #1: Yes

Reviewer #2: Yes

**4. Is the manuscript presented in an intelligible fashion and written in standard English?**
Reviewer #1: Yes

Reviewer #2: Yes

**5. Review Comments to the Author**

**Reviewer #1:** Salzano et al. conducted research to determine the preferred type of sample during the COVID-19 pandemic among individuals living in various regions across the globe. This study holds great importance and provides valuable insights for better preparedness in the face of future pandemics. Below are my observations.

- **Terms such as testing preference and testing samples are not clear. What does testing preference mean? Is it a type of specimen or a type of lab method? The manuscript needs revision in this regard.**

*Response:* We thank the reviewer for raising this concern. We have updated the text in an effort to clarify this. We have used ‘testing preferences’ to broadly cover anything in regards to the test process, whether it is the sample type used, the mode of collection (ie, self-collection or healthcare worker collected), test type, etc. In most instances we have rephrased this term to better clarify our messaging. We also reviewed all instances of the word ‘sample’ to ensure we had clarity around its use. We recognized our methods section reporting on the survey questions should have been clearer and updated this to better reflect the questions being asked in the survey. We were also sure to note that every survey included a figure which presented and explained the different sample types that we were asking about. Due to the figures that were used we were unable to include this in the supplementary data but have indicated that readers are welcome to request it from us and we are happy to also provide it to the reviewers, if helpful.

- **Abstract: Oral sample versus oral swab, are they the same? I think it should be an oral swab instead of an oral sample.**

*Response:* We apologize for the confusion regarding this terminology. We had used ‘oral sample’ in 5 instances (twice in the abstract conclusion and thrice in the discussion) as a collective term for both oral swabs and saliva samples. We have clarified this in these instances. In all other pieces of text, the exact sample type itself (either oral swab or saliva), is specifically referred to. We hope this resolves this lack of clarity on our part.

- **Line number 89 to 91: “ We aimed to better understand the public view regarding different sample types with the hope that these results could inform future surveillance planning and diagnostics development. Surveillance for which disease? Is it for COVID? Further description is needed.**

*Response:* We thank the reviewer for raising these points. We have clarified this sentence to specify that these results can apply to infectious disease more broadly - not just COVID-19.

- **It is advisable to expand all abbreviations in the first encounter.**

*Response:* We apologize for this oversight and have ensured that we have expanded upon abbreviations when first introduced.

- **Would you clarify the ‘testing method’ mentioned in Tables 3 and 4? I did not see any testing method in the table.**

*Response:* We thank the reviewer for identifying this error. We have corrected the table titles to specify that we are reporting on the preferred sample type for diagnostic testing.

- **Add some statistical analysis**

*Response:*  We thank the reviewer for raising this point. For table 1, chi-squared tests have been used to compare the distribution of respondents across regions by occupation, gender, age, and education. For table 3 and within the text, we used the Wilcoxon signed-rank test with Bonferroni correction to perform pairwise comparisons of all overall testing combinations. Within each demographic subgroup, as shown in supplementary table 20 and supplementary table 21, we used the Wilcoxon signed-rank test with Bonferroni correction to perform pairwise comparisons of all overall testing combinations.

**Reviewer #2:** The manuscript presents a significant and timely contribution to the field of public health and diagnostics, focusing on global preferences for clinical sample types used in SARS-CoV-2 detection. The study's relevance is underscored by the ongoing challenges in managing infectious diseases and the imperative for testing strategies that are both effective and acceptable to the public. Below are comments aimed at further strengthening the manuscript:

1. **While the broad geographic distribution of the survey is a strength, more detail on the survey distribution methods would enhance the reader's understanding of potential sampling biases. Specifically, clarifying how reliance on social networks and professional contacts might have skewed demographics towards healthcare professionals and those with higher education levels would be informative.**
   *Response:* Thank you for your comment. We have added clarification within the limitations section to acknowledge the reliance on personal and professional networks and how this survey method may have impacted those who saw and participated in the survey.
2. **The discussion on the reliability and public perception of saliva testing could be strengthened by incorporating more comparative data or studies on its efficacy relative to other methods.**
   *Response:* We thank the reviewer for this suggestion. Among the discussion on this, we have included references to two extensive reviews (references #12 and #15), that summarize and report on the comparative data and of studies evaluating the efficacy of saliva relative to other methods. There are a great many number of studies out there now which address this but we believe that these two in particular do a better job at discussing the broader picture surrounding this rather than simply analyzing numbers which don’t always take methodology itself into consideration. We hope the reviewer agrees that the discussion in the text and referring the reader to the broader reading materials is sufficient.
3. **The manuscript could benefit from a deeper examination of the implications of self-collected samples on testing logistics, healthcare system burden, and potential barriers to access.**
   *Response:* We appreciate this suggestion from the reviewer. We have addressed this in the discussion, including reference #24 which points to the practicalities of this in LMICs in particular. We hope that our discussion regarding this is sufficient for the reviewer.
4. **Clarify how the criteria for choosing the translated languages and how this selection might have influenced regional participation rates.**
   *Response:* We thank the reviewer for this suggestion. Unfortunately, we had very few resources to support this study so we were limited to the language abilities of those on our research team as well as close colleagues who volunteered to translate the survey for us. We have updated the text in the manuscript to reflect this.
5. **Addressing the points above could enhance the clarity, rigor, and applicability of the work.**

*Response:* We thank the reviewer for their thoughtful suggestions and hope that our edits to the manuscript have improved the readability and impact of our work and findings.

**Do you want your identity to be public for this peer review?** If you choose “no”, your identity will remain anonymous but your review may still be made public.
Reviewer #1: No

Reviewer #2: No
